# Implication of Echinochrome A in the Plasticity and Damage of Intestinal Epithelium

**DOI:** 10.3390/md20110715

**Published:** 2022-11-14

**Authors:** Ji-Su Ahn, Ye Young Shin, Su-Jeong Oh, Min-Hye Song, Min-Jung Kang, So Yeong Park, Phuong Thao Nguyen, Dang Khoa Nguyen, Hyoung Kyu Kim, Jin Han, Elena A. Vasileva, Natalia P. Mishchenko, Sergey A. Fedoreyev, Valentin A. Stonik, Yoojin Seo, Byung-Chul Lee, Hyung-Sik Kim

**Affiliations:** 1 Department of Oral Biochemistry, Dental and Life Science Institute, School of Dentistry, Pusan National University, Yangsan 50612, Republic of Korea; 2 Department of Life Science in Dentistry, School of Dentistry, Pusan National University, Yangsan 50612, Republic of Korea; 3 Education and Research Team for Life Science on Dentistry, Pusan National University, Yangsan 50612, Republic of Korea; 4 Basic Research Laboratory, Department of Physiology, College of Medicine, Smart Marine Therapeutic Center, Cardiovascular and Metabolic Disease Center, Inje University, Busan 614-735, Republic of Korea; 5 G.B. Elyakov Pacific Institute of Bioorganic Chemistry, Far-Eastern Branch of the Russian Academy of Science, 690022 Vladivostok, Russia; 6 Translational Stem Cell Biology Branch, National Heart, Lung, and Blood Institute, National Institutes of Health, Bethesda, MD 20892, USA

**Keywords:** echinochrome A, oral administration, intestinal epithelium, organoid, regeneration, revival stem cells

## Abstract

The diverse therapeutic feasibility of the sea urchin-derived naphthoquinone pigment, Echinochrome A (Ech A), has been studied. Simple and noninvasive administration routes should be explored, to obtain the feasibility. Although the therapeutic potential has been proven through several preclinical studies, the biosafety of orally administered Ech A and its direct influence on intestinal cells have not been evaluated. To estimate the bioavailability of Ech A as an oral administration drug, small intestinal and colonic epithelial organoids were developed from mice and humans. The morphology and cellular composition of intestinal organoids were evaluated after Ech A treatment. Ech A treatment significantly increased the expression of *LGR5* (~2.38-fold change, *p* = 0.009) and *MUC2* (~1.85-fold change, *p* = 0.08). Notably, in the presence of oxidative stress, Ech A attenuated oxidative stress up to 1.8-fold (*p* = 0.04), with a restored gene expression of *LGR5* (~4.11-fold change, *p* = 0.0004), as well as an increased expression of *Ly6a* (~3.51-fold change, *p* = 0.005) and *CLU* (~2.5-fold change, *p* = 0.01), markers of revival stem cells. In conclusion, Ech A is harmless to intestinal tissues; rather, it promotes the maintenance and regeneration of the intestinal epithelium, suggesting possible beneficial effects on the intestine when used as an oral medication.

## 1. Introduction

Echinochrome A (Ech A), a naphthoquinone pigment derived from sea urchins, has been clinically used for decades, since it exhibits distinctive antioxidative and anti-inflammatory abilities [1]. The medical use of Ech A was approved in Russia for cardiology and ophthalmology [2], and its therapeutic applications against cardiovascular diseases (CVDs) such as ischemic heart disease and myocardial infarction, and ocular diseases including glaucoma and degenerative diseases of the retina and cornea have been reported [3,4,5,6]. Ech A is able to alleviate the clinical symptoms by scavenging free radicals and subsequently preventing infarct formation [1,7]. Ech A is also known to suppress excessive inflammation by modulating the balance between proinflammatory cells and regulatory T cells, and directly blocking the replication of pathogens in infectious pathophysiologic circumstances [3,8,9,10,11]. Due to the broad applicability of the underlying mechanism, the therapeutic potential of Ech A for various other candidate diseases such as inflammatory bowel disease (IBD), gastric ulcer, atopic dermatitis (AD), and cystic fibrosis, has been vigorously investigated [9,10,12,13]. Moreover, it has been demonstrated that naphthoquinone pigments have low toxicity profiles in pharmacological studies in vivo and in vitro [14].

The selection of an adequate drug administration route is important to guarantee therapeutic outcomes and avoid adverse effects. In the previous studies on CVDs, Ech A was mostly administered intravenously (IV), in line with the disease features requiring immediate action of drugs after disease onset and accessibility to the target lesion [15,16]. In line, subconjunctival or peribulbar injections have been employed for the treatment of ophthalmologic diseases due to the physical proximity and resulting effectiveness of drug delivery [17,18]. In addition, several other preclinical studies using animal models took subcutaneous (SC) or intraperitoneal (IP) routes depending on the experimental design or probably for ease of experiment [8,13,19,20]. As certain degenerative diseases require repetitive treatments throughout the entire lifetime, invasive methods such as tissue-specific injections may not be suitable. With growing demands of general use for a wide spectrum of diseases, the oral administration of Ech A has been tested as a more conventional route of application. In the human study of atherosclerotic inflammation [3], it is demonstrated that orally administered Ech A has corresponding effects, regardless of the drug-delivery route. It is also shown that comparable therapeutic outcomes can be achieved by a peroral application using various preclinical animal models [12,21,22]. Yet, its safety and direct impact on the gastrointestinal (GI) tract still remain unknown.

Since the success of the first development of mini-gut [23], the intestinal organoid-culture platform has paved a way for various research studies related to the intestinal environment. This in vitro three-dimensional (3D) miniature organ consists of various cell types including intestinal stem cells (ISCs), which are crucial for self-renewal, and other differentiated cells such as Paneth cells, goblet cells, and enterocytes [24]. Of note, the heterogeneity in cellular composition enables the recapitulation in the in vivo physiology of intestinal epithelial development, which is much more relevant to in vivo biology when compared with the 2D cell-line culture system. Intestinal organoids can be further categorized into small intestine organoid (SIO) and colon organoid (CO), depending on which part of the intestine tissue was used for cultivation [24,25,26]. The generation of indefinite 3D organoids allows the study of normal intestinal physiology and the development of the pathophysiology model by co-culture with pathogens, or genetic manipulation in the manner of personalized medicine [27,28,29,30]. Therefore, naïve or genetically engineered organoids can be directly utilized for the treatment of degenerative disease or employed as a drug-testing platform using the coculturing system to assess the safety, toxicity, efficacy and the extent of use of candidate drugs [31,32].

Recently, a novel population of stem cells, revival stem cell (RSC), has been reported, which appears and contributes to the regeneration of damaged tissue in injury, especially in the intestinal epithelium [33]. RSCs are activated throughout the intestinal epithelium after acute injury, triggering the partial reprogramming of differentiated cells to repair the damaged epithelium, and are reported to be detectible and inducible in intestinal organoids [33,34]. Recent studies have demonstrated that the generation of RSCs can be induced with several regulators of signaling pathways, resulting in the promotion of regenerative responses [26,35].

In the present study, we predicted the oral bioavailability of Ech A and its potential benefits to the GI system by utilizing intestinal epithelial organoids to define the impact of Ech A on the epithelium-organizing cell subtypes of both human and mouse intestine. In particular, we determined whether the therapeutic benefits of Ech A could be preserved in the presence of overproduced reactive oxygen species (ROS), mimicking disease circumstances.

## 2. Results

### 2.1. The Effect of Ech A on Mouse Intestinal Epithelial Homeostasis

**Figure 1:**
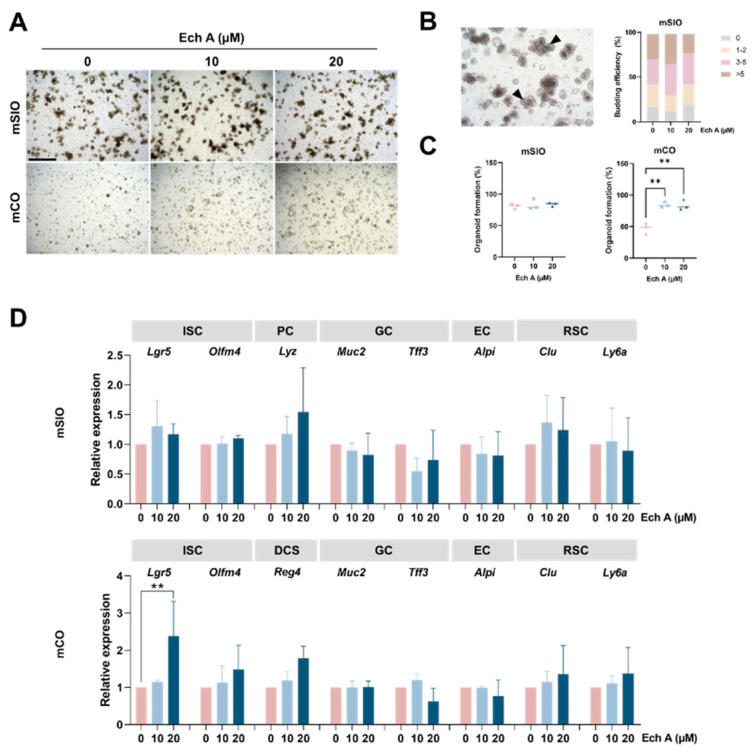
Effect of Ech A on the mouse intestinal organoids. Mouse SIOs and COs were embedded in the extracellular matrix and cultured in the presence of 10 µM and 20 µM of Ech A for 4 days. (**A**) Bright-field images of mouse intestinal organoids treated with Ech A concentration of 0, 10, 20 µM. Upper panel: mSIOs, lower panel: mCOs. (**B**) Representative image of budding structure in mSIOs (**left**) and the quantification of budding structures (**right**). Arrows indicate budding structure. (**C**) Quantification of organoid formation in mSIOs (**left**) and mCOs (**right**). (**D**) Relative marker expression of differentiated intestinal epithelial cells in mSIOs and mCOs. Ech A: Echinochrome A, mSIO: mouse small intestinal organoid, mCO: mouse colon organoid, ISC: intestinal stem cell, PC: Paneth cell, GC: goblet cell, EC: enterocyte, RSC: revival stem cell, DCS: deep crypt secretory cell. Scale bar = 1 mm. All data represent the mean ± SD of the results from three independent experiments. ** *p* < 0.01.

To understand the effect of Ech A on normal intestinal homeostasis, we established intestinal epithelial organoids from mouse small intestine and colon, and EchA was treated at concentrations of 10 µM and 20 µM. There was no toxic effect of Ech A on the organoid formation of mSIOs and mCOs (Figure 1A). Then, we determined the number of budding structures which were exhibited in the mSIOs. Upon Ech A treatment, there was no significant difference in the number of budding structures from both the control and Ech A-treated group (Figure 1B). The total number of organoids did not change in SIOs, but significantly increased in COs at both high and low concentrations of Ech A, suggesting that Ech A can promote the proliferation of colonic intestinal epithelial cells (Figure 1C). We then explored whether Ech A can regulate the differentiation of subtype cells which constitute the intestinal epithelium, including ISCs (*Lgr5*, *Olfm4*), Paneth cells (*Lyz*), deep crypt secretory cells (DCSs; *Reg4*), goblet cells (*Muc2*), enterocytes (*Alpi*), and revival stem cells (RSCs; *Clu*, *Ly6a*) (Figure 1D). Upon Ech A treatment, an increased *Lyz* expression was shown in SIOs. The gene expression of *Olfm4*, *Reg4*, *Clu*, *Ly6a* was elevated in Ech A treatment group compared with the control group in COs. Notably, the expression level of *Lgr5* (2.38-fold change, *p* = 0.009) was significantly increased in COs when Ech A was added at 20 µM concentration. Therefore, Ech A was able to partially regulate the cell composition in the intestinal epithelium and promote the formation of organoids by increasing *Lgr5* expression in mCOs.

### 2.2. The Effect of Ech A on Human Epithelial Homeostasis

**Figure 2:**
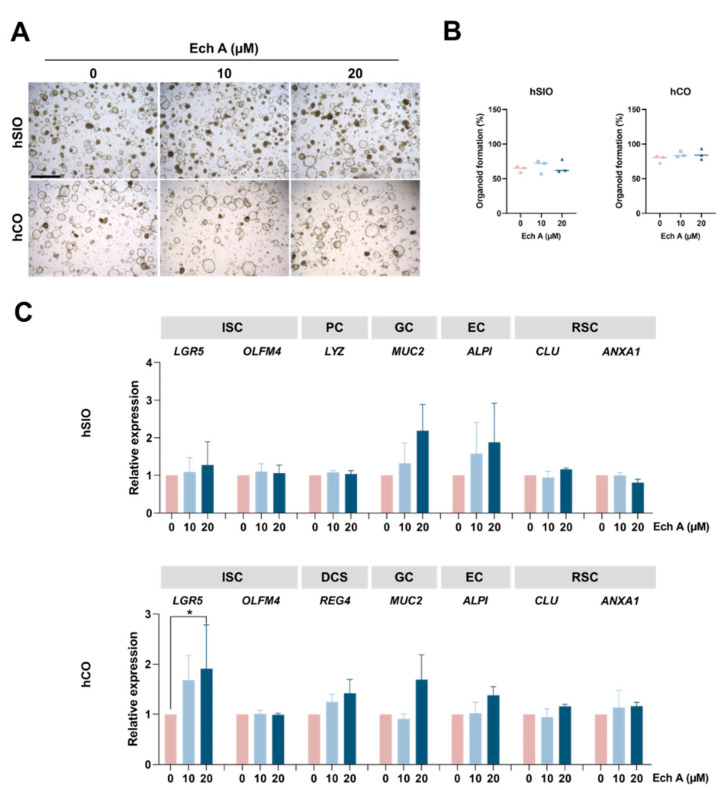
Effect of Ech A on the human intestinal organoids. Human SIOs and COs were embedded in the extracellular matrix and cultured in the presence of 10 µM and 20 µM of Ech A for 5 days. (**A**) Bright-field images of human intestinal organoids treated with Ech A concentrations of 0, 10, 20 µM. Upper panel: hSIOs, lower panel: hCOs. (**B**) Quantification of organoid formation efficiency in hSIOs (**left**) and hCOs (**right**). (**C**) Relative marker expression of differentiated intestinal epithelial cells differentiation in hSIOs and hCOs. Ech A: Echinochrome A, hSIO: human small intestinal organoid, hCO: human colon organoid, ISC: intestinal stem cell, PC: Paneth cell, GC: goblet cell, EC: enterocyte, RSC: revival stem cell, DCS: deep crypt secretory cell. Scale bar = 1 mm. All data represent the mean ± SD of the results from three independent experiments. * *p* < 0.05.

We next sought to examine the effect of Ech A on the human intestinal organoids, including SIOs and COs. Ech A treatment did not exhibit any toxic influences in hSIOs and hCOs (Figure 2A). We found that the size of organoids increased with Ech A treatment, compared with the control (Figure 2A), while the total number of organoids did not change in either SIOs or COs (Figure 2B). We further investigated the change of cellular composition in intestinal epithelium after the administration of Ech A using the markers of ISCs (*LGR5*, *OLFM4*), Paneth cells (*LYZ*), DCSs (*REG4*), goblet cells (*MUC2*), enterocytes (*ALPI*), and revival stem cells (*CLU*, *ANXA1*) (Figure 2C). The gene expression level of *MUC2*, *ALPI* was upregulated in the Ech A treatment group of hSIOs and hCOs, compared with the control. Consistent with the results in mCOs, 20 µM of Ech A markedly increased the expression of *LGR5* (1.91-fold change, *p* = 0.048) in hCOs. The expression of RSC markers exhibited no significant differences without any stimulation. These results suggest that Ech A regulates the various type of cells in the intestinal epithelium, especially increasing the stem cell population in the colon.

### 2.3. Antioxidative Ability of Ech A in Mouse Intestinal Organoids

**Figure 3:**
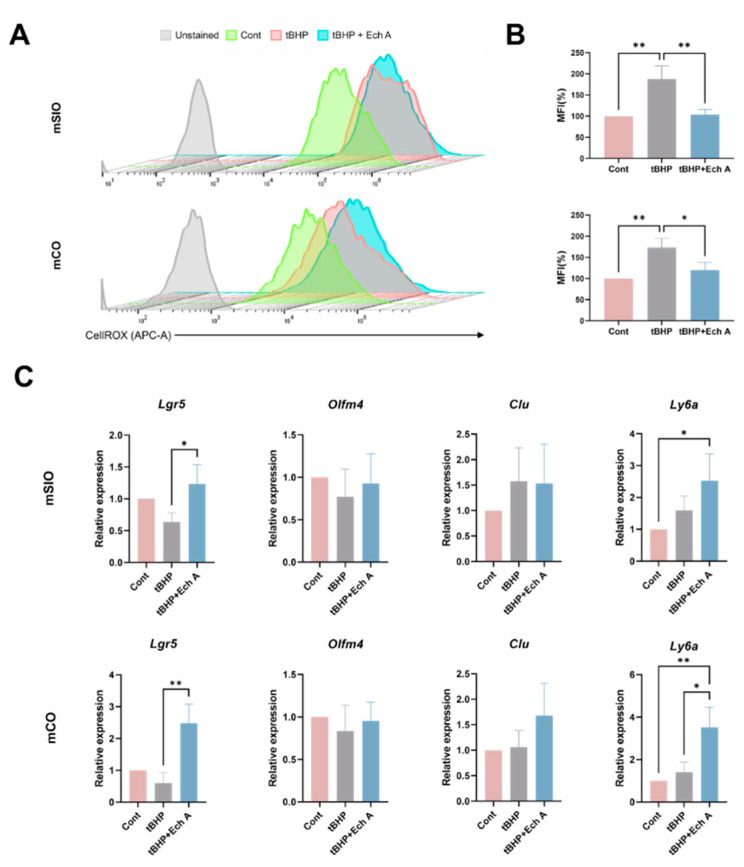
Antioxidant effect of Ech A on oxidative damage-induced mouse intestinal organoids. Organoids were treated with 250 µM tBHP in the presence or absence of 20 µM Ech A for 4 h. (**A**) Flow cytometric analysis of ROS production by using ROS dye CellROX Deep Red assay and (**B**) mean fluorescence intensities (MFI) of CellROX-labeled cells in mSIOs and mCOs. Upper panel: mSIOs, lower panel: mCOs. (**C**) Relative gene expression level of ISC (*Lgr5, Olfm4*) and RSC (*Clu, Ly6a*) markers. Ech A: Echinochrome A, tBHP: tert-Butyl hydroperoxide, mSIO: mouse small intestinal organoid, mCO: mouse colon organoid, MFI: mean fluorescence intensities. * *p* < 0.05, ** *p* < 0.01.

Ech A was previously reported to possess a potent antioxidative effect to attenuate oxidative stress [1]. Therefore, we assessed whether Ech A could exhibit antioxidative properties in the intestinal epithelial organoid by modeling the environment of oxidative stress. For this purpose, we used ROS inducer, tert-Butyl hydroperoxide (tBHP). Administration of tBHP resulted in a higher level of cellular ROS in both mSIOs (187.63%) and mCOs (173.22%) compared with the control via flow cytometric analysis (Figure 3A,B). However, upregulation of CellROX was suppressed by up to 103.68% and 120.26%, respectively, in organoids treated with both 20 µM Ech A and tBHP, suggesting that oxidative stress was resolved. To further explore the regenerative function of Ech A, we performed qPCR to examine the marker expression of ISCs and RSCs, which contribute to epithelial regeneration (Figure 3C). We found a reduced expression level of *Lgr5* upon tBHP administration in both mSIOs and mCOs, while Ech A treatment restored the expression or even increased it, compared with the control group (fold change 0.63 to 1.23 in mSIOs, *p* = 0.026; fold change 0.6 to 2.47 in mCOs, *p* = 0.032). The expression of *Olfm4* tended to decrease upon tBHP treatment, while it was not recovered by Ech A treatment. Compared with the control, tBHP treatment induced an increase in RSC marker (*Clu*, *Ly6a*) expression, which was further upregulated upon Ech A supplementation. A total of 20 µM of Ech A significantly increased the expression of *Ly6a*, compared with the control (2.51-fold change in mSIOs, *p* = 0.035; 3.51-fold change in mCOs, *p* = 0.005).

### 2.4. Antioxidative Ability of Ech A in Human Intestinal Organoids

**Figure 4:**
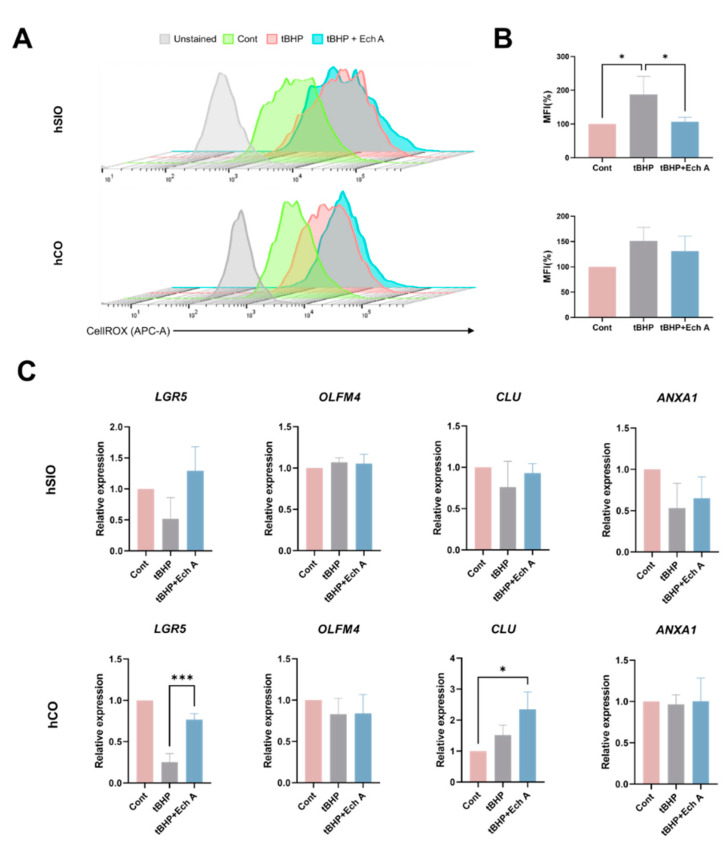
Antioxidant effect of Ech A on oxidative damage-induced human intestinal organoids. Organoids were maintained for 5 days and then treated with 250 µM tBHP in the presence or absence of 20 µM Ech A for 4 h. (**A**) Flow cytometric analysis of ROS production by using ROS dye CellROX Deep Red assay and (**B**) mean fluorescence intensities (MFI) of CellROX-labeled cells in human SIOs and COs. Upper panel: hSIOs, lower panel: hCOs. (**C**) Relative gene expression level of ISC (*LGR5, OLFM4*) and RSC (*CLU, ANXA1*) markers. Ech A: Echinochrome A, tBHP: tert-Butyl hydroperoxide, hSIO: human small intestinal organoid, hCO: human colon organoid, MFI: mean fluorescence intensities. * *p* < 0.05, *** *p* < 0.001.

Next, we analyzed the antioxidant function of Ech A in human intestinal organoids. The induction of tBHP led to an increase in ROS level (187.71% in hSIOs and 151.31% in hCOs), and ROS damage was greatly prevented by 20 µM Ech A treatment in hSIOs (106.73%) (Figure 4A,B). We then determined the marker expression to identify the effect of Ech A on the intestinal cell differentiation in the ROS-elevated status (Figure 4C). Consistent with the results from mouse intestinal organoids, a reduced expression level of *LGR5* was observed upon tBHP treatment. The administration of Ech A restored the level of *LGR5* expression (fold change 0.25 to 0.76, *p* = 0.0004) to a similar extent to the control group in hCOs. The RSC marker, *CLU* expression was upregulated by tBHP and further elevated by Ech A addition (2.35-fold change, *p* = 0.01) compared with the control in hCOs, while the reduced *ANXA1* expression upon tBHP induction in hSIOs was not changed by Ech A treatment. There was no significant difference in the expression of *OLFM4* in either hSIOs or hCOs in response to tBHP or Ech A. Taken together, our results indicate that Ech A can exhibit antioxidant scavenger properties and help epithelial regeneration by inducing the RSC population in the intestinal epithelium.

## 3. Discussion

In order to ask whether perorally administered Ech A would be likely to have clinical benefits without causing harm to intestinal tissues, we primarily tested bioavailability and latent toxicity through developed intestinal organoids, including SIOs and COs derived from both human and mouse tissues. We fortunately found that Ech A treatment of the organoids did not show any deleterious effect quantitatively or qualitatively, but rather significantly improved the development of intestinal organoids, especially COs, reflecting the provisional safety of Ech A in its oral use. Excessive ROS induction hindered the stemness of intestinal organoids, and, interestingly, Ech A treatment compensatively restored it, by stimulating not only typical ISCs, but also RSCs.

To evaluate the safety and permeability of orally administered drugs in the intestinal epithelium, a typical model of the transformed adenocarcinoma line Caco-2 monolayer transwell assay, which can mimic the epithelial barrier function, was commonly employed until recently, and contributed to many pharmacokinetic studies. However, issues have been raised concerning the fact that drug-processing mechanisms differ from primary cells, or that the interaction between the cells making up the intestinal structure is absent [36,37,38]. Recent studies in organoids have suggested versatile usages for this 3D culture system, such as natural physiology research, disease modeling, drug screening, and also treatment *per se* [27,28,29,30,31,32,39]. In particular, intestinal organoids can recapitulate the properties of the intestinal microenvironment requiring crosstalk between host cellular compartments and substances existing outside the endogenous construct, for example, infectious microorganisms (host–pathogen interaction), commensal microbiota, nutrients, metabolites, or drugs [25,40,41,42]. In this study, we introduced this readily established 3D culture system as a drug-testing platform.

We showed that Ech A treatment improved the regenerative effect of organoids. In line with the increased number, especially in colon-organoid formation, the effect is also evidenced by the upregulated expression-level of the Wnt-amplifying gene, *LGR5* (Leucine-rich repeat-containing G protein-coupled receptor 5), which is a crucial factor for the self-renewal and propagation of ISCs [43]. Contrary to our results, it is known that oxidative stress provokes *Lgr5* expression, and subsequently promotes the expansion of ISCs [44,45,46]. However, most of the papers point out that ROS-mediated *LGR5* transcription and ISCs expansion are connected to colorectal cancer initiation, not physiological turnover [44,45,47]. In fact, robust ROS production in intestinal crypts causes apoptosis and tissue dysfunction; thus, maintaining an adequate level of ROS is critical for cell proliferation and tissue renewal [46,48,49]. One potential explanation for our results is that Ech A treatment presumably modulates the optimal level of ROS for 3D organoid culture. When tBHP, the organic ROS inducer was treated, the expression level of *LGR5* remarkably decreased, while the level was restored by ROS suppression with Ech A treatment (Figure 3C and Figure 4C). We can learn from these results that optimal concentration should be carefully determined in future, considering hyperplasia concerns.

Of note is the fact that the unique cellular population in the intestinal crypt, RSCs, characterized by high clusterin (*CLU*) expression, are very rare at the level of homeostatic status. This distinct cell subset is transiently activated, and then expanded, upon physiological damage such as irradiation, loss of LGR5^+^ ISCs, and exposure to toxic chemicals, to reconstitute ISCs compartments and subsequently rehabilitate the structure and function of the normal intestinal epithelium [33,50]. In our study, we observed that RSCs, which stay at a quiescent state normally, showed no significant changes in gene-expression patterns upon Ech A treatment (Figure 1D and Figure 2C), suggesting that an influx of Ech A is not recognized as a danger signal. On the other hand, oxidative stress induction by tBHP drastically exacerbated LGR5^+^ ISCs integrity, and accordingly promoted a damage-induced cell population RSCs. Wang et al. also demonstrated that LPS-induced gut damage depleted Lgr5^+^ ISCs in the mice intestine, and this long-term loss of ISCs then triggered the production of Clu^+^ RSC in the crypts for intestinal regeneration [51]. Strikingly, the expression of RSC marker genes, *Ly6a* and *CLU*, were boosted through the action of Ech A (Figure 3C and Figure 4C). Therefore, Ech A can be expected to contribute to the homeostasis and reconstruction of the intestinal environment by controlling the activity of ISCs, as well as further increasing the activity of RSCs upon intestinal damage.

In human intestinal organoids, *MUC2* expression from goblet cells showed a tendency to increase in the presence of Ech A (Figure 2C). The mucus layer lining throughout the whole GI tract has a crucial role for protecting the body from exogenous pathogens. Intestinal goblet cells secrete various molecules, and among them mucin glycoproteins are synthesized by the activation of the *MUC2* gene. As a frontline of the host defense system, the mucus layer prevents the adhesion of the pathogen into the epithelium [52]. Similar to *MUC2* expression, we also observed that *ALPI* expression was increased in a dose-dependent manner in human intestinal organoids (Figure 2C). Intestinal alkaline phosphatase (IAP) is encoded by the *ALPI* gene, and mostly produced by enterocytes. IAP is known to detoxify bacterial ligands and upregulate intestinal tight junction proteins so that it can carry out a gut barrier function for the intestinal structure [53,54]. In addition, since the expression of IAP decreases with aging, taking Ech A has the potential benefit of overcoming age-related dysfunction [55]. Taken together, Ech A treatment facilitates the protective role of intestinal compartment cells, goblet cells and enterocytes, by stimulating the production of mucin glycoproteins and IAPs.

In our previous study, we proved that systemically administered Ech A alleviated the symptoms of experimental colitis by promoting immunomodulatory cells such as Tregs and M2 macrophages, while suppressing pro-inflammatory M1 macrophages [10]. It was reported that exposure to colitogens such as dextran sulfate sodium (DSS) impairs ISC function and in turn activates RSCs population [33]. Furthermore, *Muc2* deficiency causes spontaneous colitis, and the endogenous IAP level is lower in IBD [56,57,58]. We revealed that Ech A compensatively activated the self-renewal capacity of the intestinal structure and augmented the defensive role of goblet cells and enterocytes, in the current study. From another point of view, through these results, we may infer that the regenerative function of Ech A contributed to amelioration in the DSS colitis model.

To further understand the mechanism of Ech A action on intestinal cells, cellular signaling pathways upon Ech A exposure should be elucidated. It has been reported that Ech A treatment promotes ex vivo maintenance of CD34^+^ hematopoietic stem and progenitor cells (HSPCs), through suppression of cellular ROS and the Src-Lyn-p110δ pathway, and the activation of the PI3K/Akt pathway [59]. PI3K/Akt signaling regulates a variety of crucial cellular events such as cell proliferation and survival by regulating the activity of NF-κB signaling, which also modulates the activity of Wnt/β-catenin signaling [60]. A cross-talk between PI3K/Akt, NF-κB, and Wnt/β-catenin signaling has been considered, to regulate Lgr5^+^ ISCs [61,62]. In addition, it has been demonstrated that *MUC2* expression could be regulated by PI3K/Akt pathways [63,64]. One can envision that the regulation of stem cells by Ech A shown in the present study might be exerted through similar pathways, including the PI3K/Akt pathway. Considering that PI3K/Akt signaling can be triggered by Ech A, it would be worthwhile to investigate the activated signaling pathway in Ech A-treated IOs, in further studies. Moreover, physiologically, we previously showed that intravenous injection of Ech A exhibited a therapeutic effect in the murine colitis model, accompanied by the attenuation of intestinal epithelial damage, providing direct therapeutic evidence for Ech A in diseases [10].

Although we concisely attested to the potential peroral use of Ech A, there are some remaining limitations. The distribution and excretion of orally administered Ech A should be investigated. There are some reports that subcutaneously injected Ech A can reach the target organ through the bloodstream within hours, or be eliminated by the kidney [8,65,66]. However, pharmacokinetic and pharmacodynamic features of orally administered Ech A have not been studied yet. In order to be delivered into the intestine, exposure to stomach acid is inevitable, so investigation into the effectiveness of drugs after exposure to an acidic environment must be conducted. Previous studies on gastric ulcers have proven the therapeutic effect in an acidic environment [12], but have not fully elucidated the changes in Ech A after exposure. If acid-induced denaturation is specified, advanced drug delivery methods such as liposome encapsulation, should be considered [67,68].

## 4. Materials and Methods

### 4.1. Preparation and Treatment of Ech A

Histochrome^®^ containing 1% Ech A (6-ethyl-2,3,5,7,8-pentahydroxy-1,4-naphthoquinone, pharmaceutical, state registration number PN002362/01–2003) was provided by G.B. Elyakov Pacific Institute of Bioorganic Chemistry, FEB, RAS, Russia. Histochrome^®^ composition is 1% Ech A in a 0.9% isotonic solution (sodium carbonate and sodium chloride, 37.5 mM). The IOs were treated with Ech A at concentrations of 10 and 20 μM.

### 4.2. Mouse Intestinal Organoid Culture

To isolate the mouse intestinal crypts, the small intestine and colon tissue below the cecum were harvested, and flushed with cold PBS to remove residual substances inside. After flushing, for the SIOs, the small intestine was longitudinally incised using small scissors, and scraped using a coverslip to remove the villi. The tissue was cut into 2–4 mm pieces and washed with cold PBS. The fragments were washed by vigorous shaking until a clear supernatant was observed, approximately three times. Tissue fragments were incubated with Gentle Cell Dissociation Reagent (STEMCELL Technologies, Vancouver, BC, Canada) at room temperature for 20 min, with gentle rocking. For the COs, the colon tissue was washed three times after the flushing step, and cut into 2–4 mm pieces. The fragments were incubated in 20 µM EDTA with PBS at 37 °C for 30 min, with shaking. After the incubation, the tissues from the small intestine and colon were shaken by vortexing for crypt isolation. The crypts were filtered through a 70 μm cell strainer (BD Falcon, Franklin Lakes, NJ, USA) and centrifuged at 300× *g* for 5 min. Then, the crypts were resuspended in Advanced Dulbecco’s Modified Eagle Medium: Nutrient Mixture F-12 (DMEM/F12; Gibco, Grand Island, NY, USA), followed by centrifugation at 200× *g* for 5 min. A total of 250 crypts were resuspended in DMEM/F12 and Matrigel (Corning Life Sciences, New York, NY, USA) (1:1), and the crypt-Matrigel suspension was seeded into a 24- or 48-well plate and incubated for 30 min at 37 °C. After polymerization of the Matrigel, IntestiCult™ Organoid Growth Medium (STEMCELL Technologies, Vancouver, Canada) was added for SIOs maintenance, while a conditioned medium was used for the COs culture. The conditioned medium consisted of advanced DMEM/F12, primocin (Invivogen, San Diego, CA, USA), penicillin/streptomycin, B27, N2 (Gibco), 50 ng/mL EGF (Peprotech, Rockyhill, NJ, USA), 0.2 µM LDN193189 (MedChemExpress, Monmouth Junction, NJ, USA), 1 mM N-Acetyl cysteine (Sigma, St. Louis, MO, USA), 10 nM Y-27632 (STEMCELL technologies), 10 µM SB431542, 100 ng/mL Wnt 3a, and 500 ng/mL R-Spondin (Peprotech, Rockyhill, NJ, USA).

### 4.3. Human Intestinal Organoid Culture

All procedures involved in human samples were approved by the institutional ethical committee of the Pusan National University [IRB No. 2022_36_BR]. IOs were cultured from normal crypts of small intestine and colon samples derived from resected tissues of 3 patients with cancer. Written informed consent was obtained from each patient, according to the approved IRB protocol. Donors were fully informed and then consented, and documents regarding donor privacy are securely maintained. For the isolation of human intestinal crypts, small intestinal or colonic tissue samples were immediately incubated in the ice-cold PBS with penicillin/streptomycin, for 30 min. The surface of the mucosa was gently scraped using forceps, to remove the mucus. The tissue was cut into 2–4 mm pieces, and the fragments were washed with cold PBS three times. Then, the fragments were incubated in 10 mM EDTA at 37 °C for 20 min in the shaker. After incubation, the crypts were isolated from the tissues by vortexing, and filtered with a 70 μm cell strainer. The crypts were centrifuged at 300× *g* for 5 min, and resuspended in DMEM/F12, followed by centrifugation at 300 *g* for 5 min. Approximately 500 crypts were seeded in domes made by DMEM/F12 and Cultrex Reduced Growth Factor BME (R&D system, Abingdon, UK) (1:1) into 24- or 48-well plates, and incubated at 37 °C for 30 min. The conditioned media for human SIOs and COs was comprised of advanced DMEM/F12, primocin, penicillin/streptomycin, B27, N2, 50 ng/mL EGF, 0.2 µM LDN193189, 1mM N-Acetyl cysteine, 10 µM SB431542, 10 nM Y-27632, 100 ng/mL Wnt 3a, and 500 ng/mL R-Spondin.

### 4.4. Reverse Transcription and Quantitative Real-Time PCR (qPCR)

Organoids in the Matrigel were harvested and washed with PBS. RNA was extracted using the RNeasy Mini Kit (Qiagen, Hilden, Germany) and the concentration of RNA was measured using a spectrophotometer. The synthesis of cDNA was performed from 100 ng of total RNA using ReverTra Ace^®^ qPCR RT Master Mix (Toyobo, Osaka, Japan), according to the manufacturer’s instructions. A qPCR was performed using a cDNA template and SYBR Green reagents (Thermo Fisher Scientific, Waltham, MA, USA) containing specific primers, on an ABI 7500 real-time PCR instrument (Applied Biosystems, Carlsbad, CA, USA). Primer sequences for mouse and human samples are listed in Table 1. Triplicate technical replications were conducted, and relative expression levels were normalized, using the comparative Ct value of *Gapdh*.

### 4.5. Flow Cytometric Analysis

For flow cytometry analysis, the organoids were harvested and centrifuged at 300g for 5 min. The organoids were dissociated with tryPLE in 37 °C for 5 min, and then washed with PBS, and centrifuged. Cellular ROS was determined by incubating with 5 µM CellROX deep red (Thermo Fisher Scientific, Waltham, MA, USA) at 37 °C, for 30 min. After incubation, the samples were washed with PBS three times, and the level of cellular ROS was determined, using BD Accuri™ C6 Plus (BD Biosciences, San Jose, CA, USA). FlowJo software (Tree Star Inc., Ashland, OR, USA) was used for the data analysis. The percentage of stained cells was calculated, and the results were compared with the negative controls.

### 4.6. Statistical Analysis

All data are represented as the mean values ± standard deviation of at least three independent experiments. Statistical significance was determined using one-way ANOVA. Differences with *p*-values of less than 0.05 were considered statistically significant. Statistical analysis was performed using GraphPad Prism 9 software (GraphPad Software, Inc., La Jolla, CA, USA).

## 5. Conclusions

Echinochrome A treatment in small intestinal and colonic epithelial organoids revealed non-toxic, stem-cell supporting and anti-oxidative actions of the drug on the intestinal epithelium. Therefore, this study demonstrates that Ech A might contribute not only to the maintenance of intestinal homeostasis, but also to the protection of the intestinal epithelium against oxidative damage, and the regeneration, through the regulation of normal and regeneration-associated stem cells, suggesting beneficial and therapeutic effects for the intestine, when orally administered.

## Figures and Tables

**Table 1:** Primer sequences utilized in this study.

Gene	Forward Primer	Reverse Primer
**Mouse**		
*Alpi*	CTGCCAAGAAGCTGCAGCCCA	GGCTAGGGGTGTCTCCGGTCC
*Clu*	GCTGCTGATCTGGGACAATG	ACCTACTCCCTTGAGTGGACA
*Gapdh*	GGAAGGGCTCATGACCAC	GCAGGGATGATGTTCTGG
*Lgr5*	GGGAGCGTTCACGGGCCTTC	GGTTGGCATCTAGGCGCAGGG
*Lyz*	CGTTGTGAGTTGGCCAGAA	GCTAAACACACCCAGTCAGC
*Ly6a*	GAAAGAGCTCAGGGACTGGAGTGTT	TTAGGAGGGCAGATGGGTAAGCAA
*Muc2*	TGCCCAGAGAGTTTGGAGAG	CCTCACATGTGGTCTGGTTG
*Olfm4*	ATTCGCTATGGCCAAGGAGG	GAGGGGCCGATTCACATCAA
*Reg4*	AACCTGCCTGTGTGGATTGG	GTTCATCTCAGCGCAATGCC
*Tff3*	TAATGCTGTTGGTGGTCCTG	CAGCCACGGTTGTTACACTG
**Human**		
*ALPI*	TCCTGCCGTTGGACCTTCA	GGCCTGCTTGGTCTTCCTTA
*ANXA1*	CGA AAC AAT GCA CAG CGT CA	TCA GTG TTT CAT CCA GGG GC
*CLU*	GTTGCTTTTGCACCTACGGG	GAGCAGCAGAGTCGAGTGTT
*GAPDH*	GTCTCCTCTGACTTCAACAGCG	ACCACCCTGTTGCTGTAGCCAA
*LGR5*	GGAGTTACGTCTTGCGGGAA	CAGGCCACTGAAACAGCTTG
*LYZ*	GCCTAGCACTCTGACCTAGC	GTTCTGGCCAACTCACACCT
*MUC2*	CAGCACCGATTGCTGAGTTG	GCTGGTCATCTCAATGGCAG
*OLFM4*	TCAGCTCAACTGGAGAGGGT	GCCATAGGTGATCCGCAACT
*REG4*	TGGTTGCCAAACAGAATGCC	GGCCAGTGCCAGAGATCTAA

## Data Availability

Not applicable.

## References

[1] Lebedev A.V., Ivanova M.V., Levitsky D.O. (2005). Echinochrome, a naturally occurring iron chelator and free radical scavenger in artificial and natural membrane systems. Life Sci..

[2] Prokopov I.A., Kovaleva E.L., Minaeva E.D., Pryakhina E.A., Savin E.V., Gamayunova A.V., Pozharitskaya O.N., Makarov V.G., Shikov A.N. (2019). Animal-derived medicinal products in Russia: Current nomenclature and specific aspects of quality control. J. Ethnopharmacol..

[3] Artyukov A.A., Zelepuga E.A., Bogdanovich L.N., Lupach N.M., Novikov V.L., Rutckova T.A., Kozlovskaya E.P. (2020). Marine polyhydroxynaphthoquinone, Echinochrome A: Prevention of atherosclerotic inflammation and probable molecular targets. J. Clin. Med..

[4] Egorov E., Alekhina V., Volobueva T., Fedoreev S., Mishchenko N., Kol’tsova E. (1999). Histochrome, a new antioxidant, in the treatment of ocular diseases. Vestn. Oftalmol..

[5] Zakirova A., Lebedev A., Kukharchuk V., Mishchenko N., Fedoreev S. (1996). The antioxidant histochrome: Its effect on lipid peroxidation and the blood rheological properties in patients with unstable stenocardia. Ter. Arkh..

[6] Kim H.K., Vasileva E.A., Mishchenko N.P., Fedoreyev S.A., Han J. (2021). Multifaceted clinical effects of echinochrome. Mar. Drugs.

[7] Mischenko N., Fedoreev S., Zapara T., Ratushnyak A. (2009). Effects of histochrom and emoxypin on biophysical properties of electroexitable cells. Bull. Exp. Biol. Med..

[8] Lennikov A., Kitaichi N., Noda K., Mizuuchi K., Ando R., Dong Z., Fukuhara J., Kinoshita S., Namba K., Ohno S. (2014). Amelioration of endotoxin-induced uveitis treated with the sea urchin pigment echinochrome in rats. Mol. Vis..

[9] Yun H.R., Ahn S.W., Seol B., Vasileva E.A., Mishchenko N.P., Fedoreyev S.A., Stonik V.A., Han J., Ko K.S., Rhee B.D. (2021). Echinochrome A treatment alleviates atopic dermatitis-like skin lesions in NC/Nga mice via IL-4 and IL-13 suppression. Mar. Drugs.

[10] Oh S.-J., Seo Y., Ahn J.-S., Shin Y.Y., Yang J.W., Kim H.K., Han J., Mishchenko N.P., Fedoreyev S.A., Stonik V.A. (2019). Echinochrome A reduces colitis in mice and induces in vitro generation of regulatory immune cells. Mar. Drugs.

[11] Fedoreyev S.A., Krylova N.V., Mishchenko N.P., Vasileva E.A., Pislyagin E.A., Iunikhina O.V., Lavrov V.F., Svitich O.A., Ebralidze L.K., Leonova G.N. (2018). Antiviral and antioxidant properties of echinochrome A. Mar. Drugs.

[12] Sayed D.A., Soliman A.M., Fahmy S.R. (2018). Echinochrome pigment as novel therapeutic agent against experimentally-induced gastric ulcer in rats. Biomed. Pharmacother..

[13] Park G.-T., Yoon J.-W., Yoo S.-B., Song Y.-C., Song P., Kim H.-K., Han J., Bae S.-J., Ha K.-T., Mishchenko N.P. (2021). Echinochrome A treatment alleviates fibrosis and inflammation in bleomycin-induced scleroderma. Mar. Drugs.

[14] Shikov A.N., Pozharitskaya O.N., Krishtopina A.S., Makarov V.G. (2018). Naphthoquinone pigments from sea urchins: Chemistry and pharmacology. Phytochem. Rev..

[15] Shvilkin A., Serebriakov L., Tskitishvili O., Sadretdinov S., Kol’tsova E., Maksimov O., Mishchenko N., Novikov V., Levitskiĭ D., MIa R. (1991). Effect of echinochrom on experimental myocardial reperfusion injury. Kardiologiia.

[16] Buĭmov G., Maksimov I., Perchatkin V., Repin A., Afanas’ev S., Markov V., Karpov R. (2002). Effect of the bioantioxidant histochrome on myocardial injury in reperfusion therapy on patients with myocardial infarction. Ter. Arkh..

[17] Gakhramanov F., Kerimov K., Dzhafarov A. (2006). Use of natural antioxidants for the correction of changes in general and local parameters of lipid peroxidation and antioxidant defense system during experimental eye burn. Bull. Exp. Biol. Med..

[18] Petrova N., Rascheskov A.Y., Bolgova L., Habibullina N. (2012). Effectiveness of 0.02% pentahydroxyethylnaphtoquinone (hystochrome) in patients with active and fibrous stages of retinopathy of prematurity. Kazan Med. J..

[19] Seol J.E., Ahn S.W., Seol B., Yun H.R., Park N., Kim H.K., Vasileva E.A., Mishchenko N.P., Fedoreyev S.A., Stonik V.A. (2021). Echinochrome A Protects against Ultraviolet B-induced Photoaging by Lowering Collagen Degradation and Inflammatory Cell Infiltration in Hairless Mice. Mar. Drugs.

[20] Seo D.Y., McGregor R.A., Noh S.J., Choi S.J., Mishchenko N.P., Fedoreyev S.A., Stonik V.A., Han J. (2015). Echinochrome A improves exercise capacity during short-term endurance training in rats. Mar. Drugs.

[21] Fahmy S.R., Sayed D.A., Soliman A.M., Almortada N.Y., Aal W.E. (2019). Protective effect of Echinochrome against intrahepatic cholestasis induced by alpha-naphthylisothiocyanate in rats. Braz. J. Biol..

[22] Kuznetsova M., Lebed’Ko O., Ryzhavskii B., Mishchenko N. (2019). Effect of oral administration of echinochrome on lipopolysaccharide-induced lung injury in the immature Wistar rats. Eur. Respir. J..

[23] Sato T., Vries R.G., Snippert H.J., Van De Wetering M., Barker N., Stange D.E., Van Es J.H., Abo A., Kujala P., Peters P.J. (2009). Single Lgr5 stem cells build crypt-villus structures in vitro without a mesenchymal niche. Nature.

[24] Stelzner M., Helmrath M., Dunn J.C., Henning S.J., Houchen C.W., Kuo C., Lynch J., Li L., Magness S.T., Martin M.G. (2012). A nomenclature for intestinal in vitro cultures. Am. J. Physiol. Gastrointest. Liver Physiol..

[25] Seo Y., Oh S.-J., Ahn J.-S., Shin Y.Y., Yang J.W., Kim H.-S. (2019). Implication of Porphyromonas gingivalis in colitis and homeostasis of intestinal epithelium. Lab. Anim. Res..

[26] Regmi S., Seo Y., Ahn J.-S., Pathak S., Acharya S., Nguyen T.T., Yook S., Sung J.-H., Park J.-B., Kim J.O. (2021). Heterospheroid formation improves therapeutic efficacy of mesenchymal stem cells in murine colitis through immunomodulation and epithelial regeneration. Biomaterials.

[27] Zachos N.C., Kovbasnjuk O., Foulke-Abel J., In J., Blutt S.E., De Jonge H.R., Estes M.K., Donowitz M. (2016). Human enteroids/colonoids and intestinal organoids functionally recapitulate normal intestinal physiology and pathophysiology. J. Biol. Chem..

[28] Zhou J., Li C., Liu X., Chiu M.C., Zhao X., Wang D., Wei Y., Lee A., Zhang A.J., Chu H. (2020). Infection of bat and human intestinal organoids by SARS-CoV-2. Nat. Med..

[29] Fujii M., Clevers H., Sato T. (2019). Modeling human digestive diseases with CRISPR-Cas9–modified organoids. Gastroenterology.

[30] Dutta D., Heo I., Clevers H. (2017). Disease modeling in stem cell-derived 3D organoid systems. Trends Mol. Med..

[31] Watanabe S., Kobayashi S., Ogasawara N., Okamoto R., Nakamura T., Watanabe M., Jensen K.B., Yui S. (2022). Transplantation of intestinal organoids into a mouse model of colitis. Nat. Protoc..

[32] Crespo M., Vilar E., Tsai S.-Y., Chang K., Amin S., Srinivasan T., Zhang T., Pipalia N.H., Chen H.J., Witherspoon M. (2017). Colonic organoids derived from human induced pluripotent stem cells for modeling colorectal cancer and drug testing. Nat. Med..

[33] Ayyaz A., Kumar S., Sangiorgi B., Ghoshal B., Gosio J., Ouladan S., Fink M., Barutcu S., Trcka D., Shen J. (2019). Single-cell transcriptomes of the regenerating intestine reveal a revival stem cell. Nature.

[34] Yui S., Azzolin L., Maimets M., Pedersen M.T., Fordham R.P., Hansen S.L., Larsen H.L., Guiu J., Alves M.R.P., Rundsten C.F. (2018). YAP/TAZ-Dependent Reprogramming of Colonic Epithelium Links ECM Remodeling to Tissue Regeneration. Cell Stem Cell.

[35] Qu M., Xiong L., Lyu Y., Zhang X., Shen J., Guan J., Chai P., Lin Z., Nie B., Li C. (2021). Establishment of intestinal organoid cultures modeling injury-associated epithelial regeneration. Cell Res..

[36] Workman M.J., Gleeson J.P., Troisi E.J., Estrada H.Q., Kerns S.J., Hinojosa C.D., Hamilton G.A., Targan S.R., Svendsen C.N., Barrett R.J. (2018). Enhanced utilization of induced pluripotent stem cell–derived human intestinal organoids using microengineered chips. Cell. Mol. Gastroenterol. Hepatol..

[37] Yamashita T., Inui T., Yokota J., Kawakami K., Morinaga G., Takatani M., Hirayama D., Nomoto R., Ito K., Cui Y. (2021). Monolayer platform using human biopsy-derived duodenal organoids for pharmaceutical research. Mol. Ther. Methods Clin. Dev..

[38] Sun H., Chow E.C., Liu S., Du Y., Pang K.S. (2008). The Caco-2 cell monolayer: Usefulness and limitations. Expert Opin. Drug Metab. Toxicol..

[39] Artegiani B., Clevers H. (2018). Use and application of 3D-organoid technology. Hum. Mol. Genet..

[40] Puschhof J., Pleguezuelos-Manzano C., Martinez-Silgado A., Akkerman N., Saftien A., Boot C., de Waal A., Beumer J., Dutta D., Heo I. (2021). Intestinal organoid cocultures with microbes. Nat. Protoc..

[41] Zietek T., Rath E., Haller D., Daniel H. (2015). Intestinal organoids for assessing nutrient transport, sensing and incretin secretion. Sci. Rep..

[42] Min S., Kim S., Cho S.-W. (2020). Gastrointestinal tract modeling using organoids engineered with cellular and microbiota niches. Exp. Mol. Med..

[43] Barker N., Van Es J.H., Kuipers J., Kujala P., Van Den Born M., Cozijnsen M., Haegebarth A., Korving J., Begthel H., Peters P.J. (2007). Identification of stem cells in small intestine and colon by marker gene Lgr5. Nature.

[44] Kim S.-H., Kim K.-H., Yoo B.-C., Ku J.-L. (2012). Induction of LGR5 by H_2_O_2_ treatment is associated with cell proliferation via the JNK signaling pathway in colon cancer cells. Int. J. Oncol..

[45] Myant K.B., Cammareri P., McGhee E.J., Ridgway R.A., Huels D.J., Cordero J.B., Schwitalla S., Kalna G., Ogg E.-L., Athineos D. (2013). ROS production and NF-κB activation triggered by RAC1 facilitate WNT-driven intestinal stem cell proliferation and colorectal cancer initiation. Cell stem cell.

[46] Morris O., Jasper H. (2021). Reactive Oxygen Species in intestinal stem cell metabolism, fate and function. Free Radic. Biol. Med..

[47] Nath A., Chakrabarti P., Sen S., Barui A. (2022). Reactive oxygen species in modulating intestinal stem cell dynamics and function. Stem Cell Rev. Rep..

[48] Bhattacharyya A., Chattopadhyay R., Mitra S., Crowe S.E. (2014). Oxidative stress: An essential factor in the pathogenesis of gastrointestinal mucosal diseases. Physiol. Rev..

[49] Wu A., Yu B., Zhang K., Xu Z., Wu D., He J., Luo J., Luo Y., Yu J., Zheng P. (2020). Transmissible gastroenteritis virus targets Paneth cells to inhibit the self-renewal and differentiation of Lgr5 intestinal stem cells via Notch signaling. Cell Death Dis..

[50] Beumer J., Clevers H. (2016). Regulation and plasticity of intestinal stem cells during homeostasis and regeneration. Development.

[51] Wang S., Kai L., Zhu L., Xu B., Chen N., Valencak T.G., Wang Y., Shan T. (2021). Cathelicidin-WA Protects Against LPS-Induced Gut Damage Through Enhancing Survival and Function of Intestinal Stem Cells. Front. Cell Dev. Biol..

[52] Kim Y.S., Ho S.B. (2010). Intestinal goblet cells and mucins in health and disease: Recent insights and progress. Curr. Gastroenterol. Rep..

[53] Soares J.-B., Pimentel-Nunes P., Roncon-Albuquerque R., Leite-Moreira A. (2010). The role of lipopolysaccharide/toll-like receptor 4 signaling in chronic liver diseases. Hepatol. Int..

[54] Liu Y., Cavallaro P.M., Kim B.-M., Liu T., Wang H., Kühn F., Adiliaghdam F., Liu E., Vasan R., Samarbafzadeh E. (2021). A role for intestinal alkaline phosphatase in preventing liver fibrosis. Theranostics.

[55] Detel D., Baticic L., Varljen J. (2007). The influence of age on intestinal dipeptidyl peptidase IV (DPP IV/CD26), disaccharidases, and alkaline phosphatase enzyme activity in C57BL/6 mice. Exp. Aging Res..

[56] Tuin A., Poelstra K., de Jager-Krikken A., Bok L., Raaben W., Velders M.P., Dijkstra G. (2009). Role of alkaline phosphatase in colitis in man and rats. Gut.

[57] Ramasamy S., Nguyen D.D., Eston M.A., Nasrin Alam S., Moss A.K., Ebrahimi F., Biswas B., Mostafa G., Chen K.T., Kaliannan K. (2011). Intestinal alkaline phosphatase has beneficial effects in mouse models of chronic colitis. Inflamm. Bowel Dis..

[58] Van der Sluis M., De Koning B.A., De Bruijn A.C., Velcich A., Meijerink J.P., Van Goudoever J.B., Büller H.A., Dekker J., Van Seuningen I., Renes I.B. (2006). Muc2-deficient mice spontaneously develop colitis, indicating that MUC2 is critical for colonic protection. Gastroenterology.

[59] Park G.B., Kim M.J., Vasileva E.A., Mishchenko N.P., Fedoreyev S.A., Stonik V.A., Han J., Lee H.S., Kim D., Jeong J.Y. (2019). Echinochrome A Promotes Ex Vivo Expansion of Peripheral Blood-Derived CD34(+) Cells, Potentially through Downregulation of ROS Production and Activation of the Src-Lyn-p110delta Pathway. Mar. Drugs.

[60] Jang J., Jung Y., Chae S., Chung S.I., Kim S.M., Yoon Y. (2017). WNT/beta-catenin pathway modulates the TNF-alpha-induced inflammatory response in bronchial epithelial cells. Biochem Biophys Res. Commun.

[61] Ma B., Hottiger M.O. (2016). Crosstalk between Wnt/beta-Catenin and NF-kappaB Signaling Pathway during Inflammation. Front. Immunol..

[62] Zhao X., Ma B., Zhu H., Bai J., Liu L., Li X., Cai J., Wang B., Wang L., Pang Y. (2022). PI3K/Akt and Wnt/beta-catenin Signaling Cross-regulate NF-kappaB Signaling in TNF-alpha-induced Human Lgr5(+) Intestinal Stem Cells. Anticancer Res..

[63] El Homsi M., Ducroc R., Claustre J., Jourdan G., Gertler A., Estienne M., Bado A., Scoazec J.Y., Plaisancie P. (2007). Leptin modulates the expression of secreted and membrane-associated mucins in colonic epithelial cells by targeting PKC, PI3K, and MAPK pathways. Am. J. Physiol. Gastrointest Liver Physiol..

[64] Zhang M.M., An L.Y., Hu W.X., Li Z.Y., Qiang Y.Y., Zhao B.Y., Han T.S., Wu C.C. (2022). Mechanism of endometrial MUC2 in reproductive performance in mice through PI3K/AKT signaling pathway after lipopolysaccharide treatment. Ecotoxicol. Environ. Saf..

[65] Mishchenko N.P., Fedoreev S.A., Bagirova V. (2003). Histochrome: A new original domestic drug. Pharm. Chem. J..

[66] Talalaeva O., Mishchenko N., Briukhanov V., IaF Z., Lampatov V., Dvornikova L. (2014). Identification of histochrome metabolism products in urine for studying drug pharmacokinetics. Eksperimental’naia I Klin. Farmakol..

[67] Sokolova E.V., Menzorova N.I., Davydova V.N., Kuz’mich A.S., Kravchenko A.O., Mishchenko N.P., Yermak I.M. (2018). Effects of carrageenans on biological properties of echinochrome. Mar. Drugs.

[68] Yermak I.M., Gorbach V.I., Glazunov V.P., Kravchenko A.O., Mishchenko N.P., Pimenova E.A., Davydova V.N. (2018). Liposomal form of the echinochrome-carrageenan complex. Mar. Drugs.

